# Supplementary material for: Phospholipid-Rich DC-Vesicles with Preserved Immune Fingerprints: A Stable and Scalable Platform for Precision Immunotherapy
Source: Biomedicines. 2025 May 26;13(6):1299. doi: 10.3390/biomedicines13061299 (PMC12189087; doi:10.3390/biomedicines13061299)
Supplement: Supplementary file 1 [file biomedicines-13-01299-s001.zip › biomedicines-3637456-supplementary.pdf]

Supplementary Figure S1.

Comparative Regulatory Workflows: DC-Vesicles vs. Gene/Cell-Based Immunotherapies

Narrative:
DC-Vesicles follow a regulatory trajectory that diverges from that of gene- and cell-based therapies. As bioendogenous, non-replicative vesicle systems with well-defined structural characteristics, they may be evaluated under drug-based frameworks that emphasize documentation, analytical traceability, and platform stability. In contrast, CAR-T cells and other advanced therapy products are regulated under biologic-centric models requiring formal clinical validation, complex manufacturing processes, and extensive safety oversight. Supplementary Figure S1 presents a schematic comparison of these two regulatory paths, highlighting the reduced burden and potential acceleration offered by the non-ATMP classification route.

DC-Vesicles (Documentation-Based Pathway)		ATMP / CAR-T (Biologic Pathway)
Platform Characterization	→	Cell Origin Verification
Preclinical Dossier Submission	→	Clinical Trial Authorization
Analytical/Structural Evaluation	→	Quality Control and Vector Compliance
Adaptive Review Process	→	Stepwise Phase-Based Approval
Registration via Dossier		Full Biologic License Application

Legend:

- DC-Vesicles may be assessed under documentation-focused regulatory paradigms, which prioritize manufacturing reproducibility and mechanistic plausibility over large-scale clinical trials.
- ATMPs, including CAR-T, remain within biological agent frameworks, necessitating extended clinical, safety, and quality validation pipelines.
- This contrast reflects the strategic regulatory positioning of DC-Vesicles as precision immunotherapeutics designed for efficient translation.

Supplementary Figure S2.

Comparative Evaluation of Immunotherapy Platforms: DC-Vesicles, CAR-T Cells, and Therapeutic Exosomes

Narrative:
To contextualize the translational value of DC-Vesicles, we compared their functional and regulatory attributes with two leading immunotherapy modalities: CAR-T cells and therapeutic exosomes. Supplementary Figure S2 presents a comparative matrix that highlights distinctions in scalability, regulatory complexity, structural stability, and combination therapy compatibility. DC-Vesicles emerge as a hybrid platform—offering the immune functionality of antigen-presenting vesicles with a streamlined regulatory and manufacturing profile that supports broad clinical integration.

Attribute	DC-Vesicles (Non-ATMP)	CAR-T Cells (ATMP)	Therapeutic Exosomes
Manufacturing Model	Scalable, batch-standardized	Personalized, autologous	Variable, inconsistent yield
Cold-Chain Dependence	Stable under frozen storage	Requires cryopreservation	Sensitive to freeze–thaw
Structural Stability	High (phospholipid-enhanced)	Moderate	Low
Immunological Reproducibility	Consistent engineered markers	Donor-specific variability	Poorly characterized
Combination Therapy Potential	High (checkpoint, cytokines)	Limited by exhaustion	Experimental
Regulatory Complexity	Lower than ATMP-classified agents	High (phased clinical validation)	High (uncertain classification)
Production Burden	Moderate	Very high	Medium–High
Approval Precedent	Emerging (documentation-driven)	Established (EMA/FDA)	Sparse

Legend:

- NCE = Non-New Chemical Entity; ATMP = Advanced Therapy Medicinal Product.
- Attributes reflect typical regulatory and manufacturing characteristics for each platform.

- Data synthesized from internal development experience and published translational frameworks.

Supplementary Table S1:

Experimental Conditions for DC-Vesicle Stratification

Narrative:
DC-Vesicles were categorized into three processing groups for comparative analysis: Fresh, Concentrated, and Cryopreserved. The upstream generation protocol was consistent across all groups; differences were introduced only at the final handling stage to evaluate stability and reproducibility under distinct storage conditions.

Condition	Description
Fresh	Vesicles analyzed immediately post-harvest, without concentration or freezing.
Concentrated	Vesicles processed using standardized ultrafiltration prior to analysis.
Cryopreserved	Vesicles stored at −80 °C for 30 days before analysis.

Legend:

- All conditions were derived from a unified dendritic vesicle production workflow.
- Shared features include standardized immune activation and batch-based processing.
- Each condition was tested in biological triplicate ($n = 3$) to ensure statistical validity.

Supplementary Table S2: Complete list of identified peptides per replicate and condition.

Functional Overview of Peptide-Level Detection Across Experimental Conditions

Narrative:
This table provides a qualitative summary of peptide detection trends derived from proteomic analysis of DC-Vesicles processed under different experimental conditions. Rather than listing individual peptide sequences, the information is organized by protein family and detection behavior. This structure enables reproducibility assessment while preserving the proprietary nature of the peptide-level dataset.

Protein Category	Fresh	Concentrated	Cryopreserved	Detection Profile
Antigen-processing proteins	Broadly detected	Broadly detected	Broadly detected	High reproducibility
Redox/chaperone domains	Broadly detected	Broadly detected	Moderately detected	Partial variation under storage
Metabolic regulators	Broadly detected	Moderately detected	Variable detection	Condition-sensitive peptides
Membrane-associated motifs	Broadly detected	Broadly detected	Broadly detected	Structurally stable
Immunomodulatory fragments	Moderately detected	Broadly detected	Variable detection	Storage-responsive dynamics

Legend:

- Detection descriptors reflect condition-specific peptide-level reproducibility across biological replicates, based on internally normalized LFQ data.
- Full sequence information and spectral characteristics are archived in a validated dataset not disclosed here to protect analytical specificity.
- Peptidomic data may be shared under appropriate confidentiality terms upon justified regulatory or academic request.

Supplementary Table S3:

Summary of Immune-Relevant Protein Intensities and Variability Across Conditions

Narrative:
This table summarizes the detection and expression consistency of selected immune markers across DC-Vesicle processing conditions. The selected proteins represent key functional categories including antigen presentation, costimulation, metabolic regulation, and stress response. Values are normalized and expressed as relative detection strength (qualitative) and maximum intra-condition variability (coefficient of variation, CV%). Precise intensity values are withheld for proprietary reasons.

Protein Marker	Fresh	Concentrated	Cryopreserved	Max CV (%)	Interpretation
HLA-A	High	High	High	<12	Stable across all conditions
ICAM1	High	High	Moderate	<10	Minor reduction post-storage
CCL22	Moderate	Moderate	Moderate	<15	Uniformly detectable
NAMPT	High	Moderate	Low	<12	Storage-sensitive metabolic marker
QSOX1	High	High	Moderate	<11	Mild post-cryopreservation reduction
TIGAR	Moderate	Moderate	Variable	<18	Sensitive to freeze–thaw cycles
HSP90AB1	High	High	High	<9	Consistently preserved chaperone
LGALS9	Moderate	High	Low	<20	Potential instability in cryopreserved samples

Legend:

- Expression levels are qualitative summaries derived from normalized Razor Intensity distributions.
- CV values reflect biological triplicate reproducibility.

- "Low" indicates detectable but reduced signal; "Variable" indicates inconsistent detection across replicates.

Supplementary Table S4: Cytokine quantification before and after vesicle exposure.

Narrative:
The immunomodulatory activity of cryopreserved DC-Vesicles was evaluated based on their impact on selected cytokines representative of suppressive and pro-inflammatory immune responses. The qualitative summary in Table S3 reflects consistent directional changes across independent replicates. These shifts are indicative of a Th1-polarized response and confirm functional preservation following storage.

Cytokine	Functional Role	Observed Trend After Exposure	Interpretation
IL-10	Immune suppression	Decrease	Reduced tolerogenic signaling
TGF-β	Treg polarization	Decrease	Reversal of immunosuppressive tone
IL-12	Th1 activation	Increase	Enhanced immune priming
IFN-γ	Cytotoxic T cell support	Marked increase	Strong Th1 shift
TNF-α	Inflammatory signaling	Marked increase	Pro-inflammatory reinforcement

Legend:

- The trends reported reflect directionally consistent modulation observed across experimental replicates.
- Quantitative values (e.g., pg/mL, fold change) have been retained within a curated internal dataset to support further validation when necessary.
- Full numerical details can be made available upon justified academic or regulatory request under appropriate confidentiality safeguards.

Supplementary Table S5:

Functional Summary of Proteins Identified Across Experimental Conditions

Narrative:
To preserve the proprietary nature of the vesicle proteome while supporting

scientific transparency, Supplementary Table S4A provides a categorized overview of protein families detected across conditions. Instead of listing individual proteins, detection consistency is presented using qualitative descriptors. These data support the structural and immunological reproducibility of the DC-Vesicle platform.

Functional Category	Fresh	Concentrated	Cryopreserved	Reproducibility Tier
Antigen presentation (e.g., MHC-I)	consistently detected	consistently detected	consistently detected	High
Costimulatory / adhesion molecules	consistently detected	consistently detected	consistently detected	High
Redox / chaperone systems	consistently detected	consistently detected	moderately detected	Moderate to High
Metabolic enzymes	consistently detected	moderately detected	variably detected	Moderate
Immunomodulatory regulators	moderately detected	consistently detected	variably detected	Moderate to Low
Vesicle trafficking markers	consistently detected	consistently detected	consistently detected	High
Stress response proteins	moderately detected	moderately detected	moderately detected	Moderate

Legend:

- Terms like *consistently detected*, *moderately detected*, and *variably detected* refer to qualitative reproducibility across biological triplicates.
- Detailed protein identifiers, peptide counts, and quantification values have been retained within an internal validated dataset.
- Full proteomic results are available for regulatory or academic validation upon justified request under confidentiality terms.
- Detection terms (e.g., consistently/moderately detected) reflect reproducibility tiers across biological triplicates.
